# Percutaneous administration of allogeneic bone-forming cells for the treatment of delayed unions of fractures: a pilot study

**DOI:** 10.1186/s13287-021-02432-4

**Published:** 2021-06-26

**Authors:** Marc Jayankura, Arndt Peter Schulz, Olivier Delahaut, Richard Witvrouw, Lothar Seefried, Bruno Vande Berg, Guy Heynen, Wendy Sonnet

**Affiliations:** 1 Service d’Orthopédie – Traumatologie, Cliniques Universitaires de Bruxelles – Université Libre de Bruxelles, Hôpital Erasme, Route de Lennik 808, 1070 Brussels, Belgium; 2 Klinik für Orthopädie und Unfallchirurgie, Universität zu Lübeck, Ratzeburger Allee 160, 23568 Lübeck, Germany; 3 Labor für Biomechanik, BG Klinikum Hamburg, Bergedorfer Str. 10, 21033 Hamburg, Germany; 4 Fraunhofer Research Institution for Individualized and Cell-Based Medical Engineering, Mönkhofer Weg 239 a, 23562 Lübeck, Germany; 5 Service d’Orthopédie, Centre Hospitalier Universitaire de Charleroi, Charleroi, Belgium; 6 Department of Traumatology and Orthopaedics, Oost-Limburg Hospital, Schiepse Bos 2, Genk, Belgium; 7 Orthopedic Department, University of Wuerzburg, Wuerzburg, Germany; 8 Service de Radiologie, Cliniques Universitaires Saint-Luc, Brussels, Belgium; 9 Bone Therapeutics S.A., Gosselies, Belgium

**Keywords:** Cell therapy, Long bone, Fracture, Delayed union, Treatment, Allogeneic, Bone marrow Mesenchymal stem cells

## Abstract

**Background:**

Overall, 5–10% of fractures result in delayed unions or non-unions, causing major disabilities and a huge socioeconomic burden. Since rescue surgery with autologous bone grafts can cause additional challenges, alternative treatment options have been developed to stimulate a deficient healing process. This study assessed the technical feasibility, safety and preliminary efficacy of local percutaneous implantation of allogeneic bone-forming cells in delayed unions of long bone fractures.

**Methods:**

In this phase I/IIA open-label pilot trial, 22 adult patients with non-infected delayed unions of long bone fractures, which failed to consolidate after 3 to 7 months, received a percutaneous implantation of allogeneic bone-forming cells derived from bone marrow mesenchymal stem cells (ALLOB; Bone Therapeutics) into the fracture site (50 × 10^6^ to 100 × 10^6^ cells). Patients were monitored for adverse events and need for rescue surgery for 30 months. Fracture healing was monitored by Tomographic Union Score (TUS) and modified Radiographic Union Score. The health status was evaluated using the Global Disease Evaluation (GDE) score and pain at palpation using a visual analogue scale. The presence of reactive anti-human leukocyte antigen (HLA) antibodies was evaluated.

**Results:**

During the 6-month follow-up, three serious treatment-emergent adverse events were reported in two patients, of which two were considered as possibly treatment-related. None of the 21 patients in the per-protocol efficacy population needed rescue surgery within 6 months, but 2/21 (9.5%) patients had rescue surgery within 30 months post-treatment. At 6 months post-treatment, an improvement of at least 2 points in TUS was reached in 76.2% of patients, the GDE score improved by a mean of 48%, and pain at palpation at the fracture site was reduced by an average of 61% compared to baseline. The proportion of blood samples containing donor-specific anti-HLA antibodies increased from 8/22 (36.4%) before treatment to 13/22 (59.1%) at 6 months post-treatment, but no treatment-mediated allogeneic immune reactions were observed.

**Conclusion:**

This pilot study showed that the percutaneous implantation of allogeneic bone-forming cells was technically feasible and well tolerated in patients with delayed unions of long bone fractures. Preliminary efficacy evidence is supporting the further development of this treatment.

**Trial registration:**

NCT02020590. Registered on 25 December 2013. ALLOB-DU1, A pilot Phase I/IIa, multicentre, open proof-of-concept study on the efficacy and safetyof allogeneic osteoblastic cells (ALLOB®) implantation in non-infected delayed-union fractures.

**Supplementary Information:**

The online version contains supplementary material available at 10.1186/s13287-021-02432-4.

## Background

Bone has the remarkable intrinsic capacity to repair naturally after fracture [1–3]. The bone healing process encompasses multiple biological phenomena, including osteoconduction, osteo-induction and osteogenesis [4]. Its success is dependent on the prevailing biological and mechanical environment at the fracture site, the local blood supply, the severity of the trauma and patient-related comorbidities and habits (e.g. smoking) [4–7]. Globally, approximately 5 to 10% of fractures do not heal appropriately and result in delayed unions or non-unions, causing major disabilities for patients and leading to a huge socioeconomic burden worldwide, which is likely to increase with population ageing [3, 8, 9]. The risk of impaired fracture healing varies with the fracture location, with tibial fractures being one of the most prone to non-union [10, 11]. As stated by the diamond model, fracture healing responses are dependent on potent osteogenic cell populations, osteoconductive matrix scaffolds, growth factors and an optimum mechanical environment that provides the fracture site with adequate stability [6]. According to this concept, the use of a polytherapy approach is preferable for the management of delayed unions [5, 6]. The treatment approach should include a correction to the mechanical environment, providing the fracture site with adequate stability (fracture fixation), in addition to a local biological stimulus (bone grafting techniques, growth factors or multipotent stem cells), and should take patient-related comorbidities into account [5, 6].

Currently, the gold-standard treatment to stimulate bone healing in delayed unions and non-unions is rescue surgery with autologous bone grafts [9, 12, 13]. However, the open grafting method has some disadvantages, including the limited availability of bone autografts and osteoprogenitor cells, and the donor-site morbidity associated with autograft harvest [8, 14–17]. Bone allograft is a second option, but it has less osteo-inductive properties than autologous grafts, and it may lead to graft rejection and potential transmission of infections [8, 9, 13]. Since rescue surgery is not a harmless procedure, orthopaedic surgeons often take a watchful waiting approach, sometimes for several months, which may delay the patient’s return to a normal life and lead to a significant burden on society [18, 19]. Although there is currently no well-established, less invasive treatment approach available to foster fracture healing in patients with delayed unions, alternatives have been developed (e.g. synthetic bone substitutes, biological factors, platelet-rich plasma, biodegradable scaffolds/biomaterials in combination with osteogenic factors, electromagnetic field stimulation, low-intensity pulsed ultrasounds, cell therapy and tissue engineering products) [2, 8, 9, 12, 13, 20–22]. Previous studies have shown that bone marrow mesenchymal stem cells can differentiate into chondrocytes and osteoblasts and have the potential to increase intramembranous and endochondral ossification. Therefore, a treatment option for patients with delayed unions and non-unions could be the local implantation of bone marrow mesenchymal stem cells to replace the defective or missing osteoblastic cells [1, 5, 23–30]. Whilst injections of autologous bone marrow cells have already been used in this indication [8, 31], another alternative to accelerate an impaired fracture healing process could be the local implantation of allogeneic stem cells.

In this context, a recently developed injectable product which constituted of cultured allogeneic bone-forming cells (ALLOB; Bone Therapeutics) could be implanted locally at the fracture site to improve healing. This first-in-human study assessed the technical feasibility of the percutaneous implantation of allogeneic bone-forming cells in delayed unions of long bone fractures and provided a preliminary evaluation of their efficacy and safety during 6 months post-treatment. During a long-term follow-up, safety was further evaluated up to 30 months post-treatment.

## Patients and methods

### Study design and setting

This pilot phase I/IIa, multicentre, non-controlled, open-label, prospective study was conducted in patients with a non-infected delayed union of a long bone fracture in seven centres in Belgium and Germany between February 2014 and January 2018.

**Fig. 1:**
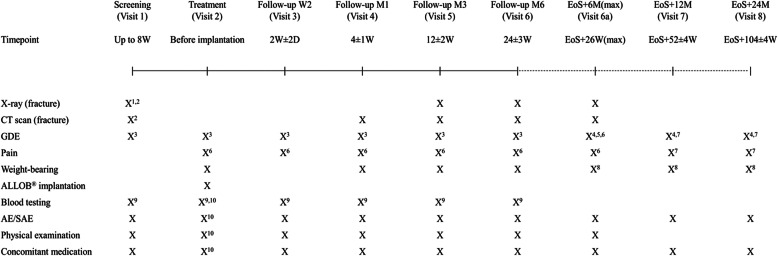
Study design. ^1^A second set of X-ray images could have been performed 1 month later during screening if pre-study images were not available; ^2^if CT scan and/or X-ray of less than 2 weeks at the time of screening were available and of sufficient quality, they could have been used as baseline images; ^3^GDE by both patient and physician; ^4^GDE by the patient only; ^5^GDE by the investigator; ^6^visual analogue scale; ^7^Likert scale; ^8^weight-bearing score (Likert scale); ^9^blood sampling for the biomarkers and auto-immunity using the blood sampling kits (in the initiation kit); ^10^evaluated before implantation and 24 and 48 h post-implantation. W, weeks; M, months; EoS, end of the study; D, days; CT, computed tomography; GDE; Global Disease Evaluation; AE, adverse event; SAE, serious adverse event. The dashed line represents the long-term safety follow-up period

Eligible patients were treated by percutaneous implantation of allogeneic bone-forming cells into the fracture site. The patients were recruited by local investigators at each site, who also performed the percutaneous implantation and the follow-up. During the study, assessments were performed via on-site visits at 2 weeks and 1, 3 and 6 months post-treatment (Fig. 1). During the long-term safety follow-up, an optional on-site visit at 12 months post-treatment could be added if judged necessary by the investigator, and patients were followed up via phone calls at 18 and 30 months post-treatment.

As this was a first-in-human study, the recruitment proceeded stepwise by blocks of 4 patients for the first 16 patients, allowing a safety data review (particularly in terms of serious treatment-emergent adverse events [TEAEs] or immunological reactions) by a Safety Monitoring Committee before the treatment of the first patient of the next block occurred. The Safety Monitoring Committee could make recommendations whether to continue or stop the trial. Moreover, as per protocol, an independent Data Safety Monitoring Board was entitled to assess the safety and efficacy results when 6-month post-treatment data for the first 16 patients were available (interim analysis). The independent Data Safety Monitoring Board could recommend whether to continue, modify or stop the trial. The study could be prematurely stopped for safety concerns, for futility if less than 4 positive cases were observed or for efficacy if at least 12 positive cases were observed.

The study was performed in accordance with the current version of the Declaration of Helsinki (Fortaleza, Brazil, October 2013) and the International Council on Harmonisation guidelines on Good Clinical Practice. The study protocol, all its amendments and the patient information sheet(s) were reviewed and approved by the appropriate Ethics Committees (CUB-ULB Erasme, Brussels, in Belgium and Universitätsklinikum Köln in Germany). The study was registered at http://www.clinicaltrials.gov (NCT02020590).

### Study population

Eligible participants were 18- to 80-year-old men or women who were diagnosed by one of the investigators with a non-infected delayed union of a long bone fracture (femur, tibia, fibula, humerus, ulna or radius) that was minimum 3 months and maximum 7 months old (± 2 weeks). Before the implantation, an independent radiologist confirmed the absence of radiological signs of progression towards healing over the last 4 weeks using conventional X-ray and/or computed tomography (CT) scans. The fracture gap—the sum of the distance between fracture edges on a mid-coronal and mid-sagittal CT reformat—was calculated by the independent radiologist as detailed in Additional file 1, and the patient was included if the fracture gap was ≤2.5 cm. On pre-implantation target bone radiographs, the modified Radiographic Union Score (mRUS) assessed by the independent radiologist had to be < 10. The Global Disease Evaluation (GDE) score assessed by the patient on a visual analogue scale (VAS) had to be ≥20 mm. A written, dated and signed informed consent was obtained from all patients or patients’ legally acceptable representatives prior to any study procedure.

Patients were excluded from the study if they had an insufficient reduction of the fracture, an insufficient fracture stability defined as osteolysis at the level of the nails/screws and/or defect and/or mobility of the osteosynthesis material, a fracture gap > 2.5 cm, a multifocal fracture, or severe nerve damage or neuropathic/neuropathic-like pain at the fracture site that may interfere with the assessments performed during the study. A complete list of exclusion criteria can be found in Additional file 2.

### Study treatment

ALLOB (Bone Therapeutics, Gosselies, Belgium) is an injectable allogeneic cell therapy product constituted of non-genetically modified viable bone-forming cells derived from ex vivo cultured bone marrow mesenchymal stem cells of the iliac crest of healthy adult donors. Allogeneic bone-forming cells are engaged towards the osteo-chondrogenic lineage whilst retaining mesenchymal stem cell properties (manuscript in preparation). The bone-forming properties of bone marrow mesenchymal stem cells were shown in a previous study where significant bone regeneration was achieved with human bone marrow-derived mesenchymal stem cell-spheroid implantation into calvarial defects in a rat model [32]. The mesenchymal stromal cells of ALLOB are cultured ex vivo under strictly controlled conditions, and allogeneic bone-forming cells are provided as a cell suspension in pre-filled syringes at a concentration of 25 × 10^6^ cells/ml.

Allogeneic bone-forming cells were injected percutaneously directly into the site of the delayed union under general or loco-regional anaesthesia. Neither scaffolds nor growth factors were used in this study. A fluoroscope was positioned over the region of the delayed union, and a 5- to 10-mm incision was made laterally through the skin and the fascia at the level of the fracture site. Under fluoroscopic control, the external trephine (Bone Therapeutics) equipped with a guiding rod was inserted manually through the subcutaneous tissue into the fracture gap between the bone fragments. After removing the guiding rod, the inner trephine was introduced into the external trephine in order to create a cavity into the fibrotic tissue in the space of the delayed union. The inner trephine was removed, and the extremity of the external trephine was connected to a syringe to push the cell suspension through the trephine into the lesion of the delayed union. The injection of the suspension was performed slowly for approximately 1 to 2 min. Finally, the trephine was washed with a rinse solution (sodium chloride 0.9%) to ensure that the entire suspension had been injected, and the syringe was removed. Gel foam or equivalent (e.g. Gelfoam®, Upjohn, USA) was pushed through the external trephine to allow clotting and closing of the hole, and the trephine was removed. The determination of the volume of suspension that a patient should receive was based on the fracture gap and the number of injection sites. A volume of 2 ml (50 × 10^6^ cells) was administered for fracture gaps of < 0.5 cm, 3 ml (75 × 10^6^ cells) for fracture gaps of ≥0.5 to ≤1 cm and 4 ml (100 × 10^6^ cells) for fracture gaps of > 1 to ≤2.5 cm. If implantation using two surgical sites was necessary as per investigator’s judgement, a total of up to 4 ml of solution was administered and two injections were performed. Patients were hospitalised for up to 48 h following the implantation procedure to allow safety follow-up.

### Study objectives

The safety endpoints included the evaluation of the occurrence of any adverse event (AE), serious AE (SAE), abnormal laboratory result and clinically relevant finding at physical examination during the entire study duration, and of potential hepatic and pulmonary secondary complications and side effects up to 6 months post-treatment.

The combined primary efficacy endpoint was the percentage of responders at 6 months post-treatment (success rate), defined as treated patients who (i) had not required rescue surgery and at the same time (ii) had an improvement in GDE score (as perceived by the patient) of at least 25% and/or an increase in Tomographic Union Score (TUS) assessed by CT of at least 2 points. The secondary efficacy endpoints were the evolution from baseline to 6 months post-treatment of radiological endpoints (based on bone changes at the fracture site using TUS and mRUS alone) and clinical endpoints (based on general health status using the GDE scores, pain at rest, during activities and at palpation for all patients, and on weight-bearing score in patients with lower extremity long bone fractures).

Alloimmunisation induced by allogeneic bone-forming cells was also evaluated.

### Data collection and analysis

During the entire study period, participants were systematically assessed for the potential occurrence of any AE and SAE using patients’ open questionnaires. AEs and SAEs were coded using the Medical Dictionary for Regulatory Activities (version 19.1). AEs occurring or worsening between the day of allogeneic bone-forming cell implantation and the last visit of the 6-month follow-up period were considered as TEAEs. Events indicative of allogeneic cell-induced reactions or ectopic bone formations were also recorded. The causal relationship between an AE/SAE and the study treatment was assessed by the investigator. At each visit, a physical examination was performed, and blood samples were collected for the evaluation of haematology, biochemistry and coagulation parameters. A chest X-ray and an ultrasonography of the liver were performed at the screening visit and at 6 months post-treatment to detect potential hepatic and pulmonary secondary complications and side effects after the allogeneic bone-forming cell implantation.

To assess fracture healing, semi-quantitative evaluation of bone production at the fracture site was performed by an independent radiologist using the TUS (at screening and 1, 3, 6 and 12 months) and mRUS (at screening and 3, 6 and 12 months). For the TUS, the four cortical areas (anterior, posterior, medial and lateral) at fracture sites were evaluated and scored on the mid-coronal and mid-sagittal CT reformats [33]. The mRUS was assessed by the same radiologist on conventional X-rays (antero-posterior and lateral) of the implanted bone by using the same callus-based score [34, 35]. Details are provided in Additional file 1.

The GDE scores assessed by the patient and the physician were used to evaluate the patient’s general health at screening, before implementation, at 2 weeks and at 1, 3, 6, 12, 18 and 30 months. It uses a 100-mm VAS where 0 means the best possible and 100 the worst possible health status. Pain was evaluated before implementation, at 2 weeks and at 1, 3, 6, 12, 18 and 30 months by the patient at rest and during activities, and by the investigator or the study nurse at palpation using a 100-mm VAS where 0 means “no pain” and 100 “extreme pain”. For patients with lower limb fractures, the functionality of the affected limb was evaluated by the weight-bearing score using a Likert scale before implementation and at 1, 3, 6, 12, 18 and 30 months. Patients were asked to place only as much weight as they felt comfortable (as tolerated) on the injured limb (based on pain feeling).

Blood samples to test for alloimmunisation were collected at baseline and during the 6-month follow-up period (at 24 and 48 h, 2 weeks and 1, 3 and 6 months post-treatment). The antibody responses induced by allogeneic bone-forming cells were assayed by the Luminex method to detect a panel of reactive anti-human leukocyte antigen (HLA) antibodies [36, 37].

### Statistical analysis

Using the two-stage Fleming method with a type I error rate α of 5% and a statistical power of 80%, the target sample size was 32 treated patients to demonstrate a success rate above 30% when considering a desirable success rate of at least 70%. Two analysis sets were defined: the safety population, which included all treated patients, and the per-protocol efficacy population, which included all patients of the safety population without any major protocol deviation.

Quantitative variables were summarised using descriptive statistics (number of observed values, mean, standard deviation [SD], median, first and third quartiles, minimum and maximum values). Categorical data were described using counts and percentages. Missing data were not taken into account in the calculation of percentages. Two-sided tests were performed at a 5% level of significance, except for efficacy analyses where a 10% level of significance was used, following a Schoenfeld approach [38].

Safety analyses were mainly descriptive. Percentages and 95% confidence intervals (CIs) were computed for qualitative variables. For efficacy analyses, percentages of responders were calculated with 90% CIs using the Clopper-Pearson method. The normality of the quantitative variables (TUS, mRUS, GDE, pain and weight-bearing scores) was tested using a Shapiro-Wilk test (normality assumed if p > 0.10). The percentages of patients with anti-HLA antibodies were described using counts and percentages. Statistical analyses were performed using SAS software (SAS Institute Inc., Cary, NC, USA) version 9.3 and above.

## Results

### Study population and treatment

The study enrolled 25 patients and was stopped by the Data Safety Monitoring Board when the analysis of the 6-month visit data in the first 16 patients indicated that no safety concerns had been identified and that the pre-defined efficacy criterion had been reached (≥12 successes were observed).

**Fig. 2:**
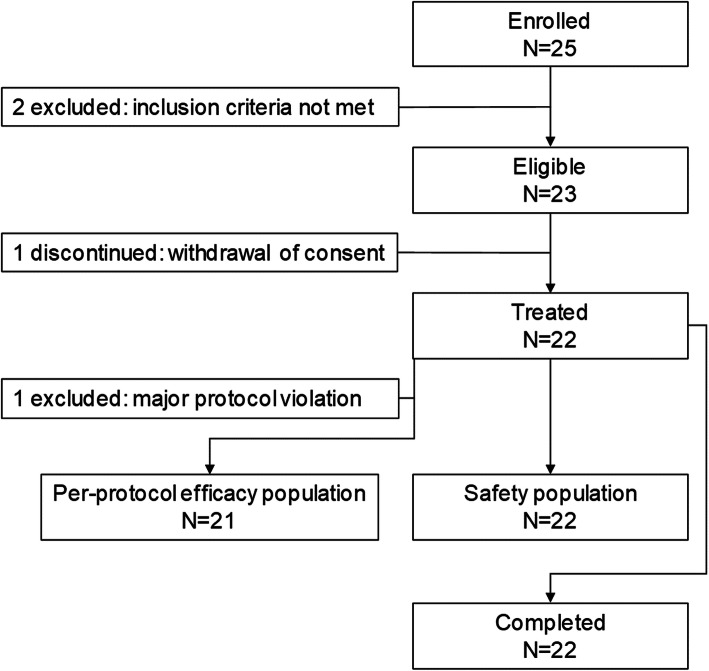
Flow of participants through the study. N, number of participants

Of the 25 enrolled patients, 23 were eligible at screening and 22 received the treatment (Fig. 2). Two enrolled patients were excluded due to a screening failure (two inclusion criteria [i.e. patient diagnosed with a non-infected delayed union of a long bone of minimum 3 months and maximum 7 months, and an mRUS < 10] were not met for each patient). All 23 eligible patients presented at least one protocol deviation before the end of the 6-month follow-up period (mainly tests not done as per protocol, non-compliance with study procedures or presence of ineligibility criteria). One of these protocol deviations led to the withdrawal of one patient from the study after treatment; this patient was excluded from the per-protocol efficacy population but was included in the safety population.

In the safety population, the mean age at enrolment was 47.3 years, 59.1% of patients were male and all patients were Caucasian (Table 1). The mean body mass index was 26.7 kg/m^2^. The percentage of current smokers was 31.8% (versus 15% in the general Belgian population in 2018 [39]). Physical examination and laboratory analyses showed no signs of malnutrition, hypoproteinemia or anaemia.

**Table 1:** Demographic characteristics (safety population)

Characteristics	
Total number of participants	22
Age (years), mean (SD)	47.3 (13.96)
Male gender, n (%)	13 (59.1)
BMI (kg/m^2^), mean (SD)	26.73 (4.676)
Ethnic origin, n (%)	
Caucasian	22 (100.0)
Smoking habits, n (%)	
Never smoked	11 (50.0)
Current smoker	7 (31.8)
Previous smoker	4 (18.2)

*SD*, standard deviation; *n (%)*, number (percentage) of participants in a given category; *BMI*, body mass index

Among the 22 treated patients, the most frequently fractured bones were the tibia (8 [36.4%] patients) and the humerus (5 [22.7%] patients) (Table 2). Fracture gaps of < 0.5 cm, ≥0.5 to ≤1 cm and > 1 to ≤2.5 cm were reported for 13 (59.1%), 7 (31.8%) and 2 (9.1%) patients, respectively. Fractures were closed in 18 (81.8%) and open in 4 (18.2%) patients. Fracture orientation was oblique in 8 (36.4%), transverse in 6 (27.3%), spiral in 6 (27.3%) and comminuted in 2 (9.1%) patients. Osteosynthesis was internal in 19 (86.4%) patients and external in 3 (13.6%) patients (Table 2).

**Table 2:** Fracture characteristics and osteosynthesis at the screening visit (safety population)

Fracture characteristics	
Total number of participants	22
Fractured bone, n (%)	
Tibia	8 (36.4)
Humerus	5 (22.7)
Femur	3 (13.6)
Ulna	3 (13.6)
Fibula	2 (9.1)
Radius	1 (4.5)
Fracture level, n (%)	
Diaphysis	19 (86.4)
Metaphysis	2 (9.1)
Epiphysis	1 (4.5)
Open/closed fracture at onset, n (%)	
Closed	18 (81.8)
Open	4 (18.2)
Type of fracture, n (%)	
Oblique fracture	8 (36.4)
Transverse fracture	6 (27.3)
Spiral fracture	6 (27.3)
Comminuted fracture	2 (9.1)
Mean fracture gap, n (%)	
< 0.5 cm	13 (59.1)
≥0.5–≤1 cm	7 (31.8)
> 1–≤2.5 cm	2 (9.1)
Type of osteosynthesis, n (%)	
Internal	19 (86.4%)
External	3 (13.6%)
If internal, n (%)	
Plate	13 (68.4%)
Nail	4 (21.1%)
Nail/metal ring	1 (5.3%)
Nail/screw	1 (5.3%)

*n (%)*, number (percentage) of participants in a given category

The mean time from fracture to implantation was 6.6 months, ranging from 3.9 to 7.9 months (Table 3). All patients received the entire volume of allogeneic bone-forming cells as prescribed by the investigator: 8 (36.4%) patients received 2 ml, 1 (4.5%) patient received 3 ml and 13 (59.1%) patients received 4 ml. Implantation was performed at two sites in 3/14 (21.4%) patients with information available. The procedure was performed under general anaesthesia in 20 patients.

**Table 3:** Allogeneic bone-forming cell implantation procedure (safety population)

Procedure characteristics	
Total number of participants	22
Time from fracture to implant (months)	
Mean (SD)	6.59 (1.159)
Median (min–max)	6.9 (3.9–7.9)
Prescribed dose	
2 ml	8 (36.4)
3 ml	1 (4.5)
4 ml	13 (59.1)
Allogeneic bone-forming cell implantation using two different surgical approaches, n (%)*	
No	11 (78.6)
Yes	3 (21.4)
Type of anaesthesia, n (%)	
General	20 (90.9)
Loco-regional	2 (9.1)
Length of stay at the hospital (days), mean (SD)	1.68 (0.78)

*SD*, standard deviation; *min*, minimum; *max*, maximum; *n (%)*, number (percentage) of participants in a given category. *N = 14 participants with available information

### Safety results

The mean (± SD) duration of follow-up for the 22 treated patients was 5.6 ± 0.4 months. Three serious TEAEs were reported in two patients: medical device site infection for the first patient, and angioedema and urticaria for the second patient (Table 4). The serious TEAEs in the latter patient, which started 1 week after and was reported 4 weeks after the implantation, could have been related to a hypersensitivity reaction to pantoprazole and enoxaparin sodium that were administered to the patient a few days post-treatment. Whilst the investigator classified these serious TEAEs as not related to the treatment, the sponsor reported them as suspected unexpected serious adverse reaction (SUSAR) as a precautionary measure because specific anti-HLA antibodies were detected. Further details are provided in Additional file 3. No immediate hypersensitivity reactions to the treatment, no ectopic bone formations, no hardware failures, no tumours and no deaths were reported.

**Table 4:** Any treatment-emergent adverse events up to the study end (safety population)

Type of adverse event	Participants (N = 22)	Adverse events (N* = 65)
	% (95% CI)	n
**Any**	81.8 (59.7–94.8)	56
**TEAEs related to the treatment°**	13.6 (2.9–34.9)	3
Oedema peripheral	4.5 (0.1–22.8)	1
Arthralgia	4.5 (0.1–22.8)	1
Pruritus	4.5 (0.1–22.8)	1
**TEAEs related to the study procedure°**	27.3 (10.7–50.2)	9
Oedema peripheral	4.5 (0.1–22.8)	1
Procedural pain	22.7 (7.8–45.4)	5
Arthralgia	4.5 (0.1–22.8)	1
Dysesthesia	4.5 (0.1–22.8)	1
Pruritus	4.5 (0.1–22.8)	1
**Serious TEAEs**	9.1 (1.1–29.2)	3
Medical device site infection	4.5 (0.1–22.8)	1
Angioedema	4.5 (0.1–22.8)	1
Urticaria	4.5 (0.1–22.8)	1

*N*, number of participants; N*, adverse events occurring up to month 6; *%*, percentage of participants in a given category; *CI*, confidence interval; *n*, number of adverse events; *TEAE*, treatment-emergent adverse event

°Related as judged by the investigator

During the 6-month follow-up period, 18 (81.8%) patients experienced a total of 56 TEAEs, with 41 (73.2%), 13 (23.2%) and 2 (3.6%) of these events being mild, moderate and severe in intensity, respectively. The most frequently reported TEAE was procedural pain, which was reported in 5 (22.7%) patients. The entire list of TEAEs is given in Additional file 4. Based on the investigator’s judgement, three non-serious TEAEs, which were moderate in intensity, were considered related to the treatment (oedema peripheral [n = 1], arthralgia [1] and pruritus [1]), and nine non-serious TEAEs were considered related to the procedure (procedural pain [n = 5], oedema peripheral [1], arthralgia [1], pruritus [1] and dysesthesia [1]) (Table 4).

Fracture radiographs did not raise safety concerns throughout the 6-month follow-up period (data not shown). No abnormalities were observed on chest X-ray, liver ultrasound, vital signs and physical examination (data not shown).

### Efficacy results

#### Treatment response

No patients required rescue surgery within 6 months post-treatment (Table 5). An improvement of GDE score of ≥25% was reported for 16/21 (76.2%) patients and an increase in TUS of at least 2 points for 16/21 (76.2%) patients; all patients met at least one of these criteria (primary endpoint definition together with no rescue surgery). At 18 months post-treatment, 2/21 (9.5%) patients had needed rescue surgery due to pseudarthrosis. No additional rescue surgeries were needed up to 30 months post-treatment.

**Table 5:** Response rate at 6 months after allogeneic bone-forming cell implantation (per-protocol efficacy population)

Response characteristics	
Total number of participants	21
Responders, % (90% CI)	100 (86.71–100)
Rescue surgery, n (%)	
No	21 (100)
Improvement of GDE score by ≥25%, n (%)	
Yes	16 (76.2)
No	5 (23.8)
Increase of TUS by ≥2 points, n (%)	
Yes	16 (76.2)
No	5 (23.8)

*CI*, confidence interval; *n (%)*, number (percentage) of participants in a given category; *GDE*, Global Disease Evaluation; *TUS*, Tomographic Union Score

#### Radiological endpoints

**Fig. 3:**
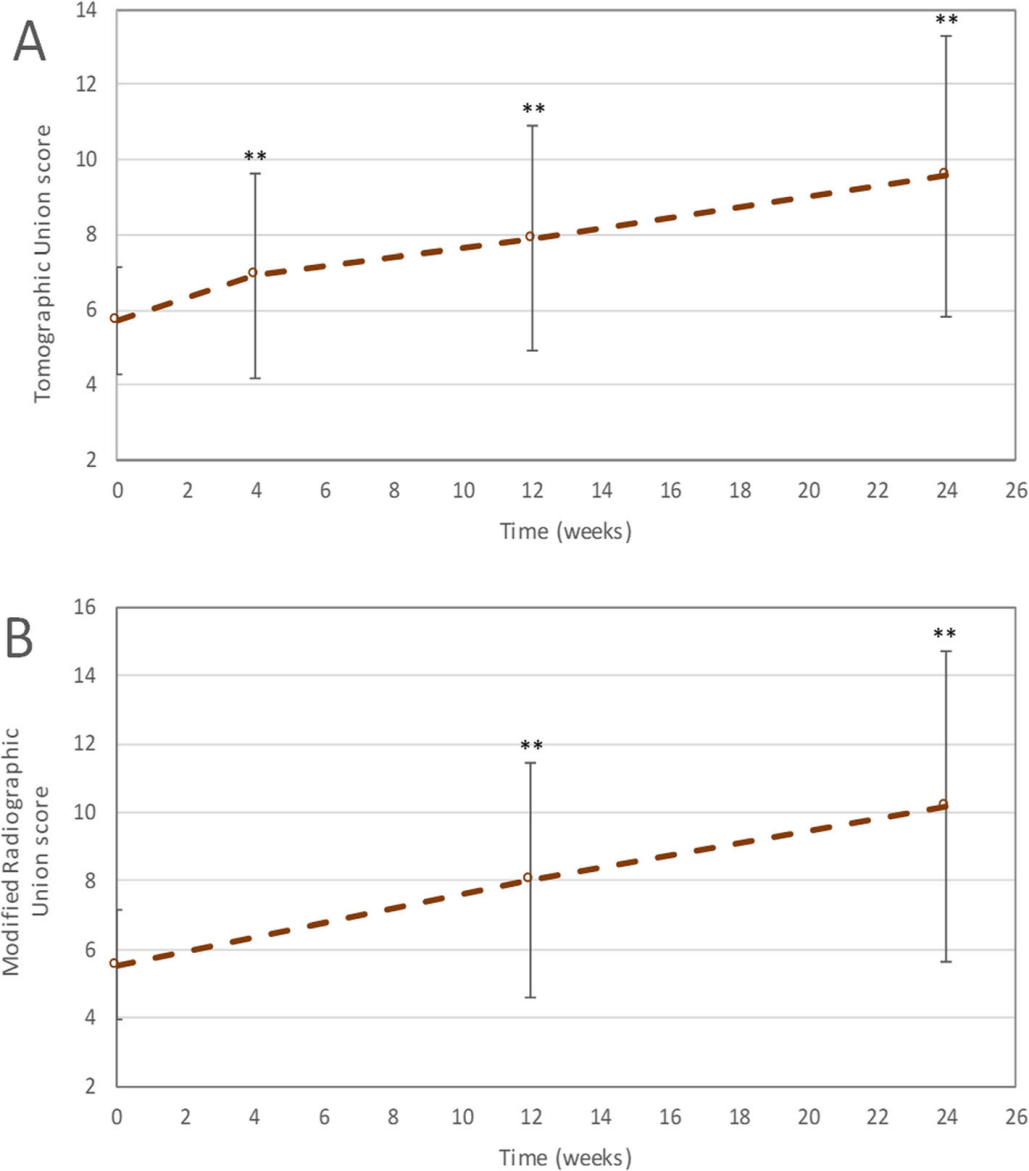
Evolution of mean A total TUS (CT scan) and B mRUS (X-ray) (per-protocol efficacy population). TUS, Tomographic Union Score; CT, computed tomography; mRUS, modified Radiographic Union Score. Error bars represent the standard deviation. **Significantly higher mean total TUS/mRUS than the mean total TUS/mRUS at baseline (least square means analysis with time and baseline as fixed effects provided p-values ≤ 0.01)

At baseline, the TUS (mean ± SD) was 5.7 ± 1.5. At 1, 3 and 6 months post-treatment, TUS were 6.9 ± 2.7, 7.9 ± 3.0 and 9.6 ± 3.8, respectively (Fig. 3A). The TUS increased by a mean of 3.8 points at 6 months post-treatment compared to baseline (*p* < 0.01). Out of the 21 patients included in the per-protocol efficacy population, 5 patients showed no radiological improvement in terms of TUS (less than 2-point increase) (Table 5). CT scans of the fracture at baseline, 3 and 6 months post-treatment are shown for two patients in Additional file 5.

At baseline, the mRUS (mean ± SD) was 5.6 ± 1.6. At 3 and 6 months post-treatment, mRUS were 8.0 ± 3.4 and 10.2 ± 4.5, respectively (Fig. 3B). The mRUS increased by a mean of 4.6 points at 6 months post-treatment compared to baseline (*p* < 0.01).

#### Clinical endpoints

**Fig. 4:**
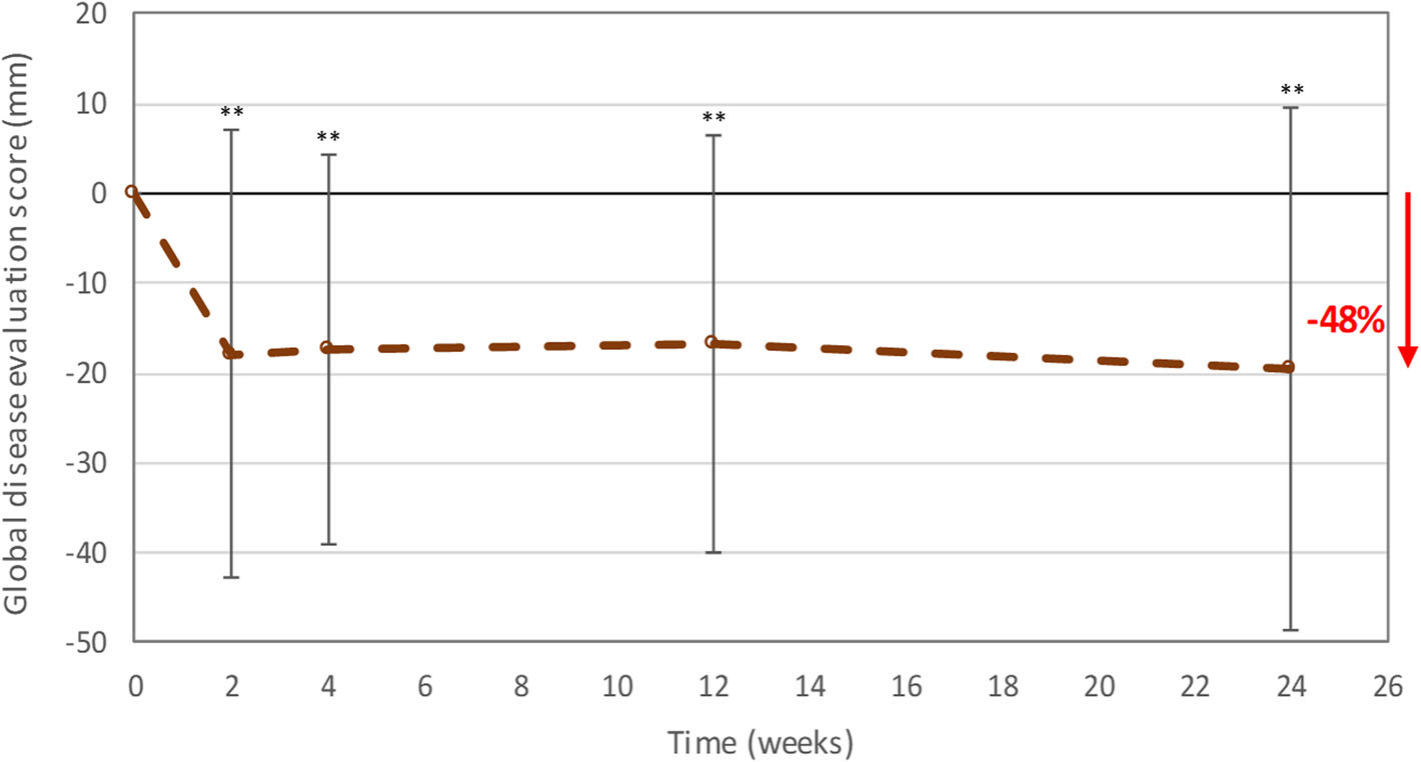
Change from baseline in mean GDE score evaluated by the patient (per-protocol efficacy population). GDE, Global Disease Evaluation. Error bars represent the standard deviation. **Significantly lower mean GDE score than the mean GDE score at baseline (least square means analysis with time and baseline as fixed effects provided p-values ≤ 0.01)

The GDE scores (mean ± SD) evaluated by the patients were 40.8 ± 18.3 mm at baseline, 23.4 ± 23.6 mm at 2 weeks and 21.3 ± 21.5 mm at 6 months post-treatment. The health status of patients measured by the GDE score improved by an average of 48% at 6 months post-treatment compared to baseline (Fig. 4). The same tendency was observed when the GDE score was evaluated by the physician (Additional file 6).

**Fig. 5:**
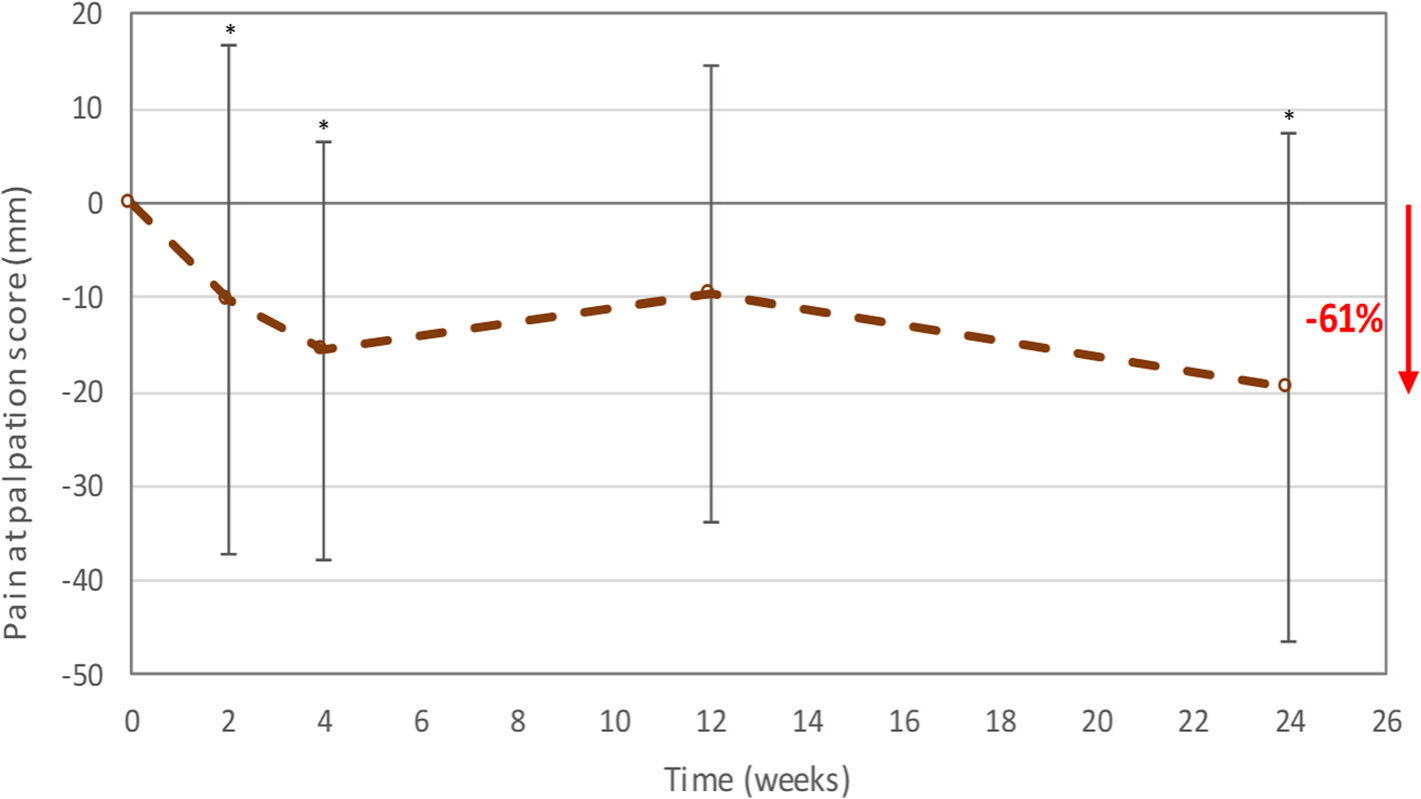
Change from baseline in mean pain at palpation score (per-protocol efficacy population). Error bars represent the standard deviation. *Significantly lower mean pain at palpation score than the mean pain at palpation score at baseline (least square means analysis with time and baseline as fixed effects provided p-values ≤ 0.05)

The pain at palpation scores (mean ± SD) were 32.1 ± 25.0 mm at baseline, 20.9 ± 22.3 mm at 2 weeks and 11.9 ± 21.8 mm at 6 months post-treatment. Pain at palpation at the fracture site was reduced by on average 61% at 6 months post-treatment compared to baseline (Fig. 5). The same tendency was observed when the pain at rest and during activities was evaluated by the patients (Additional file 7).

**Fig. 6:**
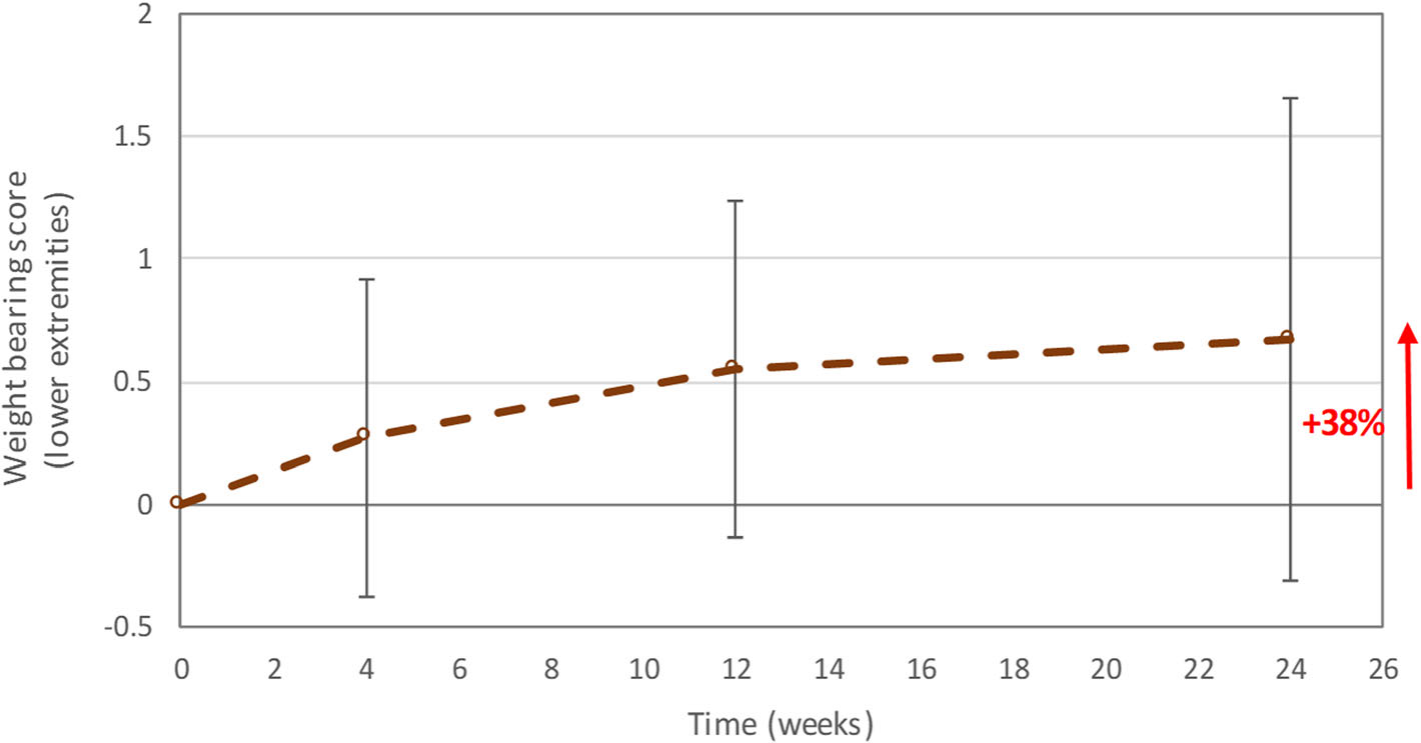
Mean weight-bearing score for long bones of the lower extremities (per-protocol efficacy population). Error bars represent the standard deviation

Most of the 12 patients with lower extremity long bone fractures had a weight-bearing score of “one” (3 patients) or “two” (6 patients) at baseline, which tended to increase over the follow-up period (Fig. 6). At 6 months post-treatment, most patients had a weight-bearing score of “two” (2 patients) or “three” (8 patients). The weight-bearing score improved by on average 38% at 6 months post-treatment compared to baseline.

### Anti-HLA antibody immune response

The proportion of patients with anti-HLA antibodies increased from 8/22 (36.4%) at baseline to 13/22 (59.1%) at 6 months post-treatment (Table 6). All anti-HLA class I/II antibody-positive patients at 6 months post-treatment had donor-specific anti-HLA antibodies, mainly against HLA class I.

**Table 6:** Overview of anti-HLA antibodies detected before and after allogeneic bone-forming cell implantation (safety population)

Anti-HLA antibody	Baseline (*N* = 22) n (%)	Week 2 (*N* = 22) n (%)	Month 1 (*N* = 22) n (%)	Month 3 (*N* = 21) n (%)	Month 6 (*N* = 22) n (%)
**Anti-HLA-positive patients**					
Anti-HLA class I antibodies (Luminex I)	4 (18.2)	5 (22.7)	9 (40.9)	12 (57.1)	13 (59.1)
Anti-HLA class II antibodies (Luminex II)	6 (27.3)	6 (27.3)	6 (27.3)	7 (33.3)	8 (36.4)
Anti-HLA class I/II antibodies (Luminex I/II)	8 (36.4)	8 (36.4)	10 (45.5)	12 (57.1)	13 (59.1)
**ALLOB-specific anti-HLA-positive patients**					
Anti-HLA class I antibodies (Luminex I)	4 (18.2)	4 (18.2)	9 (40.9)	12 (57.1)	13 (59.1)
Anti-HLA class II antibodies (Luminex II)	3 (13.6)	3 (13.6)	4 (18.2)	4 (19.0)	3 (13.6)
Anti-HLA class I/II antibodies (Luminex I/II)	5 (22.7)	5 (22.7)	9 (40.9)	12 (57.1)	13 (59.1)

*HLA*, human leukocyte antigen; *N*, number of participants; *n (%)*, number (percentage) of participants with indicated anti-HLA antibodies; *ALLOB*, allogeneic bone-forming cells

## Discussion

In this first-in-human study, we demonstrated the safety and technical feasibility of the implantation of allogeneic bone-forming cells in patients with 3- to 8-month-old, stable, non-infected delayed unions of long bone fractures without issues related to osteosynthesis material, and we provided preliminary estimates of the efficacy of this approach.

This study showed that the implantation of allogeneic bone-forming cells can be done without severe side effects in this population. No failed procedures, technical problems, tumours, ectopic/heterotopic ossifications, dystrophy nor immediate hypersensitivity reactions were reported. Three serious TEAEs were reported in two patients (medical device site infection, and angioedema and urticaria). In one of these two patients, who fully recovered, angioedema and urticaria were reported 4 weeks after the procedure as a hypersensitivity reaction without any established causal relationship with the implanted allogeneic bone-forming cells. This hypersensitivity reaction was classified as a SUSAR by the sponsor as a precautionary measure because donor-specific anti-HLA antibodies were detected in the patient’s blood. However, these events could also have been related to other medications given to the patient (pantoprazole and enoxaparin sodium).

The current study yielded preliminary imaging and clinical information on treatment efficacy. Semi-quantitative analysis of baseline and follow-up CT scans and X-ray images demonstrated an increase in scores considered indicative of bone production. The TUS and mRUS increased by 3.8 and 4.6 points, respectively, after 6 months of follow-up compared to baseline. Although the mRUS has been validated [34, 35], the assessment of fracture healing on radiographs remains challenging [40, 41]. The TUS was used because CT scans are more sensitive, have a higher correlation with callus mechanical properties and give a more global view of the fracture [33, 40]. Although it was not a validated threshold to demonstrate bone healing, 76.2% of patients had at least a 2-point improvement in TUS at 6 months post-treatment. From a clinical perspective, some improvements in global health, in pain (at rest, at palpation and during activities) and in weight-bearing score as compared to baseline were noticed as early as 2 weeks to 1 month after the implantation and persisted up to 6 months. None of the patients who were treated in the current study needed rescue surgery during the first 6 months post-treatment, but two patients underwent rescue surgery within 30 months post-treatment due to pseudarthrosis. Of note, these patients presented risk factors, such as smoking and broken osteosynthesis material, that could explain the appearance of pseudarthrosis. Together, these results provide preliminary information concerning the efficacy of allogeneic bone-forming cell implantation for the treatment of delayed unions of fractures. Due to the between-study differences in patient population and evaluation criteria, our results are difficult to compare with those of published trials evaluating other percutaneous treatment approaches for patients with delayed unions of fractures, such as the implantation of autologous bone marrow cells [42, 43], autologous concentrated bone marrow cells [44], in vitro cultured autologous mesenchymal stem cells [45] or culture-expanded autologous mesenchymal stromal cells with biomaterials [16]. Therefore, additional well-designed studies that include a control group are needed to allow a better assessment of the efficacy of this treatment.

In line with previously published studies using allogeneic mesenchymal stem cells or their derivatives, blood samples of 59.1% of our patients contained human-specific anti-HLA antibodies, which were either present before or developed after treatment [46–48]. In our study, more than one-third of patients had pre-existing anti-HLA antibodies, which is higher than anti-HLA antibody levels previously measured in volunteer blood donors (1.0–4.4% of men and nulliparous women, and 24.0–30.4% of parous women) [49, 50]. Although no clinical events (safety or efficacy) related to the detection of these anti-HLA antibodies, no treatment-related hypersensitivity AEs and no treatment-mediated allogeneic immune reactions were observed, the clinical significance of the detection of de novo anti-HLA antibodies in the current context is unknown [47, 51], and the persistence and functionality of these antibodies (complement fixation) remain to be investigated carefully in larger studies.

The preliminary results obtained in this study are encouraging since progression towards healing was observed in delayed unions of long bone fractures aged up to 8 months, suggesting that the implantation of allogeneic bone-forming cell might enhance bone formation in these patients. In mice, allogeneic bone-forming cells were shown to act by a direct stimulation of host cells to produce bone (intramembranous ossification) through paracrine factors (osteo-induction properties) and also by the production of bone from allogeneic bone-forming cells origin through endochondral ossification (intermediate cartilage phase) to replace the damaged bone (osteogenic properties) (unpublished results). The implantation of allogeneic bone-forming cells could help to improve the cellular environment in a disturbed bone healing process, for which low levels of progenitor cells as well as systemic mesenchymal and osteogenic cell pool defects were observed [5]. However, further studies are needed to evaluate to what extent our preliminary efficacy results are associated with an early healing response induced by the treatment and to determine the proportion of patients in whom healing would occur naturally without treatment.

The limitations of this study included the small sample size, the variety of bones, fracture patterns and fixation devices, the absence of a control group treated with locally implanted autologous stem cells and the open-label design of the study. Other drawbacks were the absence of evaluation of neuromuscular functions and inflammatory factors during the follow-up as well as the lack of long-term evaluation of human-specific anti-HLA immune responses and of potential problems associated with these antibodies (including the risks in case of second injection). Hence, the results of this study should be interpreted with caution and further investigations are required to eliminate these shortcomings.

## Conclusions

This pilot study showed that in patients with delayed unions of fractures of various long bones, the direct percutaneous implantation of allogeneic bone-forming cells at the fracture site was technically feasible and well tolerated and provided preliminary evidence for its potential efficacy. These results support the further evaluation of this approach.

## Supplementary Information

**Additional file 1.** Radiological evaluation.

**Additional file 2.** Exclusion criteria.

**Additional file 3.** Patient with serious treatment-emergent adverse events due to a hypersensitivity.

**Additional file 4.** Treatment-emergent adverse events up to end of study (safety population).

**Additional file 5.** CT-scans of the fracture at baseline, 3 and 6 months post-treatment for patients with (A) a closed oblique fracture of the right tibia with a gap < 0.5 cm and (B) a closed transverse fracture of the left humerus with a gap < 0.5 cm.

**Additional file 6.** Change from baseline in mean Global Disease Evaluation score evaluated by the physician (per protocol efficacy population). Error bars represent the standard deviation. * significantly lower mean Global Disease Evaluation score than the mean Global Disease Evaluation score at baseline (least square means analysis with time and baseline as fixed effects provided p-values ≤0.05).

**Additional file 7.** Change from baseline in mean (A) pain at rest score and (B) pain during activities score (per protocol efficacy population). Error bars represent the standard deviation. * significantly lower mean pain at rest/during activities score than the mean pain at rest/during activities score at baseline (least square means analysis with time and baseline as fixed effects provided p-values ≤0.05).

## Data Availability

The datasets used and/or analysed during the current study are available from the corresponding author on reasonable request.

## Abbreviations

ALLOB: Allogeneic bone-forming cells
AE: Adverse event
CI: Confidence interval
CT: Computed tomography
GDE: Global Disease Evaluation
HLA: Human leukocyte antigen
mRUS: Modified Radiographic Union Score
SAE: Serious adverse event
SD: Standard deviation
SUSAR: Suspected unexpected serious adverse reaction
TEAE: Treatment-emergent adverse event
TUS: Tomographic Union Score
VAS: Visual analogue scale
