# Supplementary material for: Percutaneous administration of allogeneic bone-forming cells for the treatment of delayed unions of fractures: a pilot study
Source: Stem Cell Res Ther. 2021 Jun 26;12:363. doi: 10.1186/s13287-021-02432-4 (PMC8235864; doi:10.1186/s13287-021-02432-4)
Supplement: Supplementary file 1 — Additional file 1. Radiological evaluation. [file 13287_2021_2432_MOESM1_ESM.docx]

**Additional file 1. Radiological evaluation**

Tomographic Union Score (TUS)

The TUS is a semi-quantitative scoring system that can be applied on mid-sagittal and mid-coronal CT reformats of a fracture to assess bone formation. A score is attributed to the two fracture sites on the mid-sagittal reformat (anterior and posterior cortices) and two fracture sites on the mid-coronal reformat (medial and lateral cortices) according to the following features : Score 1: no callus; score 2: discontinuous callus; score 3 continuous, not remodelled callus; score 4: remodelled continuous callus with sharp cortical contours. The TUS ranges from 4 to 16.

Fracture interlines were calculated as the sum of the four distances measured between the fracture edges at each of the same four fracture sites on the sagittal (anterior and posterior) and coronal (medial and lateral) reformats.

Modified Radiographic Union Score (mRUS)

The mRUS, is a semi-quantitative scoring system that can be applied on lateral and AP radiographs to assess bone formation [[25](#_ENREF_25), [38](#_ENREF_38)]. A cortical score from 1 to 4 was given to each cortical segment of the fracture site: score 1: lack of callus; score 2: non-bridging callus; score 3: bridging callus; score 4: remodelled bridging callus. For each bone fracture, the mRUS value was calculated by adding the four cortical scores. The minimum mRUS value is 4 (presence of a fracture line and absence of callus on any of the cortices) and the maximum value is 16 (remodelled continuous callus at all four fracture sites).
