# Supplementary material for: Percutaneous administration of allogeneic bone-forming cells for the treatment of delayed unions of fractures: a pilot study
Source: Stem Cell Res Ther. 2021 Jun 26;12:363. doi: 10.1186/s13287-021-02432-4 (PMC8235864; doi:10.1186/s13287-021-02432-4)
Supplement: Supplementary file 2 — Additional file 2. Exclusion criteria. [file 13287_2021_2432_MOESM2_ESM.docx]

**Additional file 2. Exclusion criteria**

Patients meeting any of the following criteria were excluded from the study:

- Current symptoms and/or signs related to the disease under study

- Fracture interline >2.5 cm, as defined by the Independent Radiologist

- Insufficient reduction of the fracture (upon the Independent Radiologist or the Investigator’s judgement)

- Insufficient fracture stability defined as osteolysis at the level of the nails/screws and/or defect and/or mobility of the osteosynthesis material at physical examination, as assessed by the investigator

- Osteosynthesis material revision or surgery (i) performed less than 2 months from the screening visit at the fracture site or (ii) performed less than 4 weeks from the screening visit at distance of the fracture site.

- Active bone infection (at site)

- Femoral neck fracture, if the femur is the target bone of the study

- Multifocal fracture (e.g., more than one fracture site on the studied bone)

- Symptomatic delayed/non-union fracture on the neighbouring bone, as judged by the Investigator

- Severe nerve damage and/or neuropathic/neuropathic-like pain at fracture site, that may interfere with assessment during the study, as appreciated by the Investigator

- Severe tendon lesion (e.g., rupture or enthesopathy) at fracture site, that may interfere with assessment during the study, as appreciated by the Investigator

- Current or previous diagnoses, signs and/or symptoms

- Positive serology for human immunodeficiency virus (defined as positive Anti-HIV 1 and/or 2 and/or positive Polymerase Chain Reaction)

- Active hepatitis B (defined as positive HBs Ag and/or positive PCR)

- Active hepatitis C (defined as positive Anti-HCV and/or positive PCR)

- Global sepsis

- Renal impairment, defined as serum creatinine > 2 mg/dl or 176 μmol/l

- Hepatic impairment, defined as alanine aminotransferase or aspartate aminotransferase ≥3 times the upper normal limit

- Poorly controlled diabetes mellitus (defined as haemoglobin A1c >8%)

- Known allergy to gentamicin

- History of hypersensitivity to human biological material including blood and blood derived products, documented clinically or by laboratory tests

- Current or past history of solid or haematological neoplasia

- History of organ or bone marrow transplantation

- Active auto-immune disease (e.g., sclerodermia, Sjögren syndrome, lupus)

- Any concomitant disease that could interfere with the evaluation of efficacy, as judged by the Investigator, including but not limited to local or metabolic bone diseases

- Life expectancy less than 6 months

- Current or previous treatment

- Patients who have previously been treated with ALLOB®

- Participation in another clinical study involving a pharmacological treatment within 3 months prior to screening

- Current (or within 1 month of screening) treatment with calcitonin, raloxifen, teriparatide, and/or strontium ralenate

- Current (or within 6 months of screening) illicit drug abuse (as per local law)

- Safety aspects concerning female subjects of childbearing potential

- Pregnancy

- Breast-feeding

- Woman not willing or able to use a reliable contraceptive method for at least 6 weeks prior to screening and during the whole study period. Reliable contraceptive methods include orally administered hormonal contraceptives, surgical intervention (e.g., tubal ligation), and intrauterine device

- Woman with positive urine pregnancy tests at Visits #1 and/or #2

- Other exclusion criteria

- Body Mass Index of 35 kg/m^2^ or greater

- Unable to undergo general anaesthesia or a surgical intervention
