# Supplementary material for: Percutaneous administration of allogeneic bone-forming cells for the treatment of delayed unions of fractures: a pilot study
Source: Stem Cell Res Ther. 2021 Jun 26;12:363. doi: 10.1186/s13287-021-02432-4 (PMC8235864; doi:10.1186/s13287-021-02432-4)
Supplement: Supplementary file 3 — Additional file 3. Patient with serious treatment-emergent adverse events due to a hypersensitivity. [file 13287_2021_2432_MOESM3_ESM.docx]

**Additional file 3. Patient with serious treatment-emergent adverse events due to a hypersensitivity**

Four weeks after the implantation, this patient was hospitalised due to the development of skin abnormalities, which started one week after the implantation. They were diagnosed as urticaria and angioedema (Quincke's oedema). A few days after surgery, this patient had received drugs known to be associated with these events (pantoprazol and enoxaparin sodium). Urticaria and angioedema are reported as rare side effects in the Summary of Product Characteristics of pantoprazol. Urticaria is reported as a common side effect in the Summary of Product Characteristics of enoxaparin sodium.

The patient was negative for anti-HLA antibodies at baseline and became positive 4 weeks post-treatment, developing antibodies against HLA type I determinants from the donor (types A3 and A24) and against non-donor determinant (type A23). Although these events were classified as not related to the treatment by the investigator, the Sponsor reported them as Suspected Unexpected Serious Adverse Reaction (SUSAR) as a precautionary measure.
