# Supplementary material for: Percutaneous administration of allogeneic bone-forming cells for the treatment of delayed unions of fractures: a pilot study
Source: Stem Cell Res Ther. 2021 Jun 26;12:363. doi: 10.1186/s13287-021-02432-4 (PMC8235864; doi:10.1186/s13287-021-02432-4)
Supplement: Supplementary file 4 — Additional file 4. Treatment-emergent adverse events up to end of study (safety population). [file 13287_2021_2432_MOESM4_ESM.docx]

**Additional file 4. Treatment-emergent adverse events up to end of study (safety population)**

| **Treatment-emergent adverse event** | **Participants (N=21)** | **Adverse events (N=65)** |
| --- | --- | --- |
|  | **% (95% CI)** | **n** |
| Any | 81.8 (59.7–94.8) | 56 |
| Ear and labyrinth disorders | 4.5 (0.1–22.8) | 1 |
| Vertigo | 4.5 (0.1–22.8) | 1 |
| Gastrointestinal disorders | 13.6 (2.9–34.9) | 5 |
| Abdominal pain | 4.5 (0.1–22.8) | 1 |
| Nausea | 4.5 (0.1–22.8) | 1 |
| Oesophagitis | 4.5 (0.1–22.8) | 1 |
| Oral dysesthesia | 4.5 (0.1–22.8) | 1 |
| Vomiting | 4.5 (0.1–22.8) | 1 |
| General disorders and administration site conditions | 22.7 (7.8–45.4) | 6 |
| Chills | 4.5 (0.1–22.8) | 1 |
| Implant site pain | 4.5 (0.1–22.8) | 1 |
| Malaise | 4.5 (0.1–22.8) | 1 |
| Medical device discomfort | 4.5 (0.1–22.8) | 1 |
| Oedema peripheral | 4.5 (0.1–22.8) | 1 |
| Peripheral swelling | 4.5 (0.1–22.8) | 1 |
| Immune system disorders | 4.5 (0.1–22.8) | 1 |
| Seasonal allergy | 4.5 (0.1–22.8) | 1 |
| Infections and infestations | 27.3 (10.7–50.2) | 8 |
| Bronchitis | 4.5 (0.1–22.8) | 1 |
| Gastroenteritis | 4.5 (0.1–22.8) | 1 |
| Gastrointestinal infection | 4.5 (0.1–22.8) | 1 |
| Medical device site infection | 4.5 (0.1–22.8) | 2 |
| Influenza | 4.5 (0.1–22.8) | 1 |
| Sinusitis | 4.5 (0.1–22.8) | 1 |
| Urinary tract infection | 4.5 (0.1–22.8) | 1 |
| Injury, poisoning and procedural complications | 31.8 (13.9–54.9) | 7 |
| Procedural pain | 22.7 (7.8–45.4) | 5 |
| Fall | 9.1 (1.1–29.2) | 2 |
| Investigations | 9.1 (1.1–29.2) | 2 |
| Body temperature increased | 4.5 (0.1–22.8) | 1 |
| C-reactive protein increased | 4.5 (0.1–22.8) | 1 |
| Metabolism and nutrition disorders | 9.1 (1.1–29.2) | 2 |
| Vitamin d deficiency | 9.1 (1.1–29.2) | 2 |
| Musculoskeletal and connective tissue disorders | 22.7 (7.8–45.4) | 6 |
| Arthralgia | 4.5 (0.1–22.8) | 1 |
| Back pain | 4.5 (0.1–22.8) | 1 |
| Bone pain | 4.5 (0.1–22.8) | 1 |
| Myalgia | 4.5 (0.1–22.8) | 1 |
| Pain in extremity | 4.5 (0.1–22.8) | 1 |
| Tendonitis | 4.5 (0.1–22.8) | 1 |
| Nervous system disorders | 18.2 (5.2–40.3) | 4 |
| Dizziness | 4.5 (0.1–22.8) | 1 |
| Dysesthesia | 4.5 (0.1–22.8) | 1 |
| Headache | 4.5 (0.1–22.8) | 1 |
| Sciatica | 4.5 (0.1–22.8) | 1 |
| Respiratory, thoracic and mediastinal disorders | 4.5 (0.1–22.8) | 1 |
| Nasal inflammation | 4.5 (0.1–22.8) | 1 |
| Skin and subcutaneous tissue disorders | 13.6 (2.9–34.9) | 10 |
| Angioedema | 4.5 (0.1–22.8) | 2 |
| Blister | 4.5 (0.1–22.8) | 1 |
| Pruritus | 4.5 (0.1–22.8) | 1 |
| Urticaria | 4.5 (0.1–22.8) | 6 |
| Surgical and medical procedures | 4.5 (0.1–22.8) | 1 |
| Removal of internal fixation | 4.5 (0.1–22.8) | 1 |
| Vascular disorders | 9.1 (1.1–29.2) | 2 |
| Phlebitis | 4.5 (0.1–22.8) | 1 |
| Thrombophlebitis superficial | 4.5 (0.1–22.8) | 1 |

N, number of participants; %/n, percentage/number of participants in given category; CI, confidence interval
