# Supplementary figures and images for: Percutaneous administration of allogeneic bone-forming cells for the treatment of delayed unions of fractures: a pilot study
Source: Stem Cell Res Ther. 2021 Jun 26;12:363. doi: 10.1186/s13287-021-02432-4 (PMC8235864; doi:10.1186/s13287-021-02432-4)

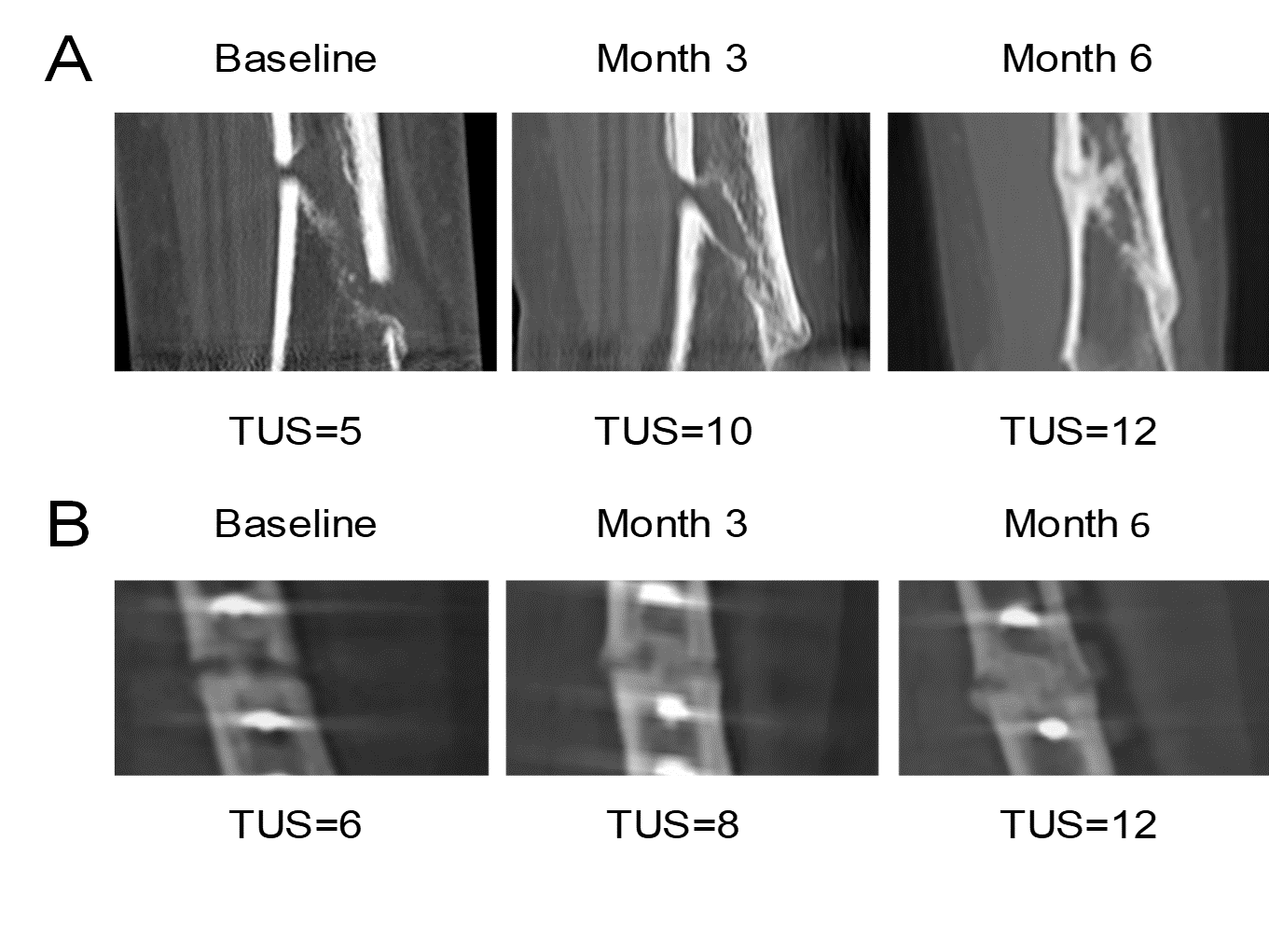

Supplement: Supplementary file 5 — Additional file 5. CT-scans of the fracture at baseline, 3 and 6 months post-treatment for patients with (A) a closed oblique fracture of the right tibia with a gap < 0.5 cm and (B) a closed transverse fracture of the left humerus with a gap < 0.5 cm. [file 13287_2021_2432_MOESM5_ESM.png]

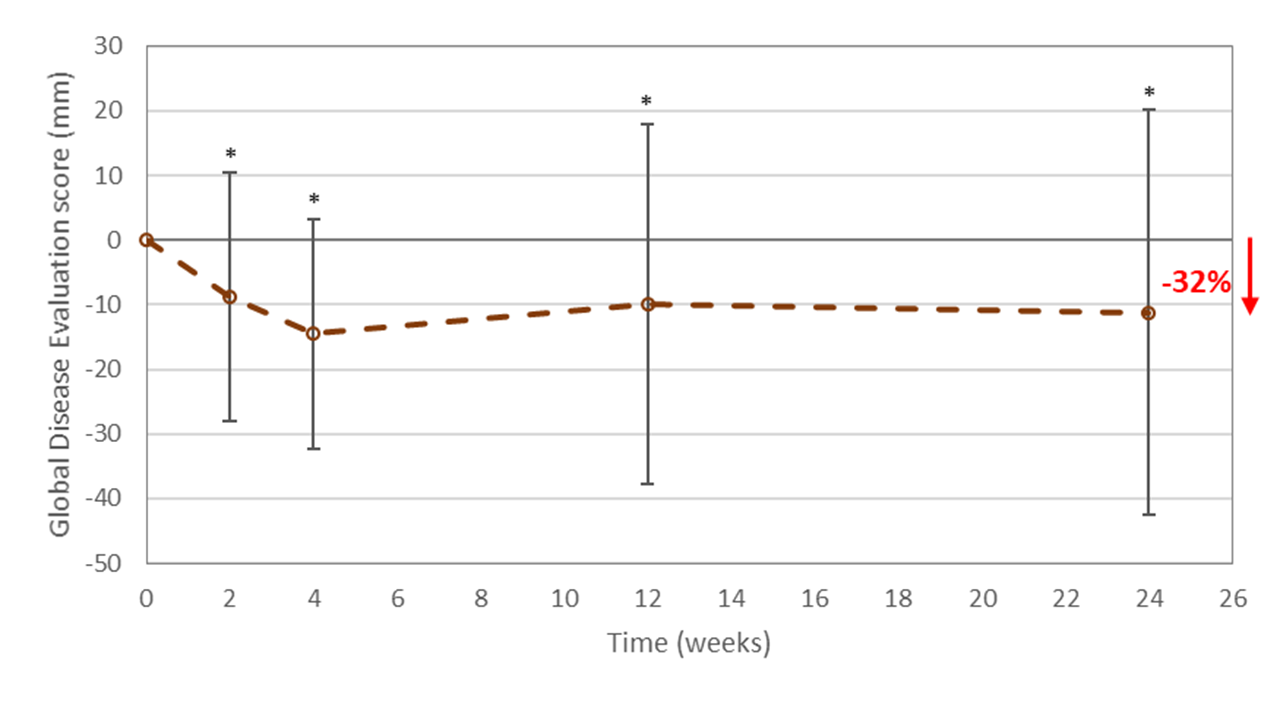

Supplement: Supplementary file 6 — Additional file 6. Change from baseline in mean Global Disease Evaluation score evaluated by the physician (per protocol efficacy population). Error bars represent the standard deviation. * significantly lower mean Global Disease Evaluation score than the mean Global Disease Evaluation score at baseline (least square means analysis with time and baseline as fixed effects provided p-values ≤0.05). [file 13287_2021_2432_MOESM6_ESM.png]

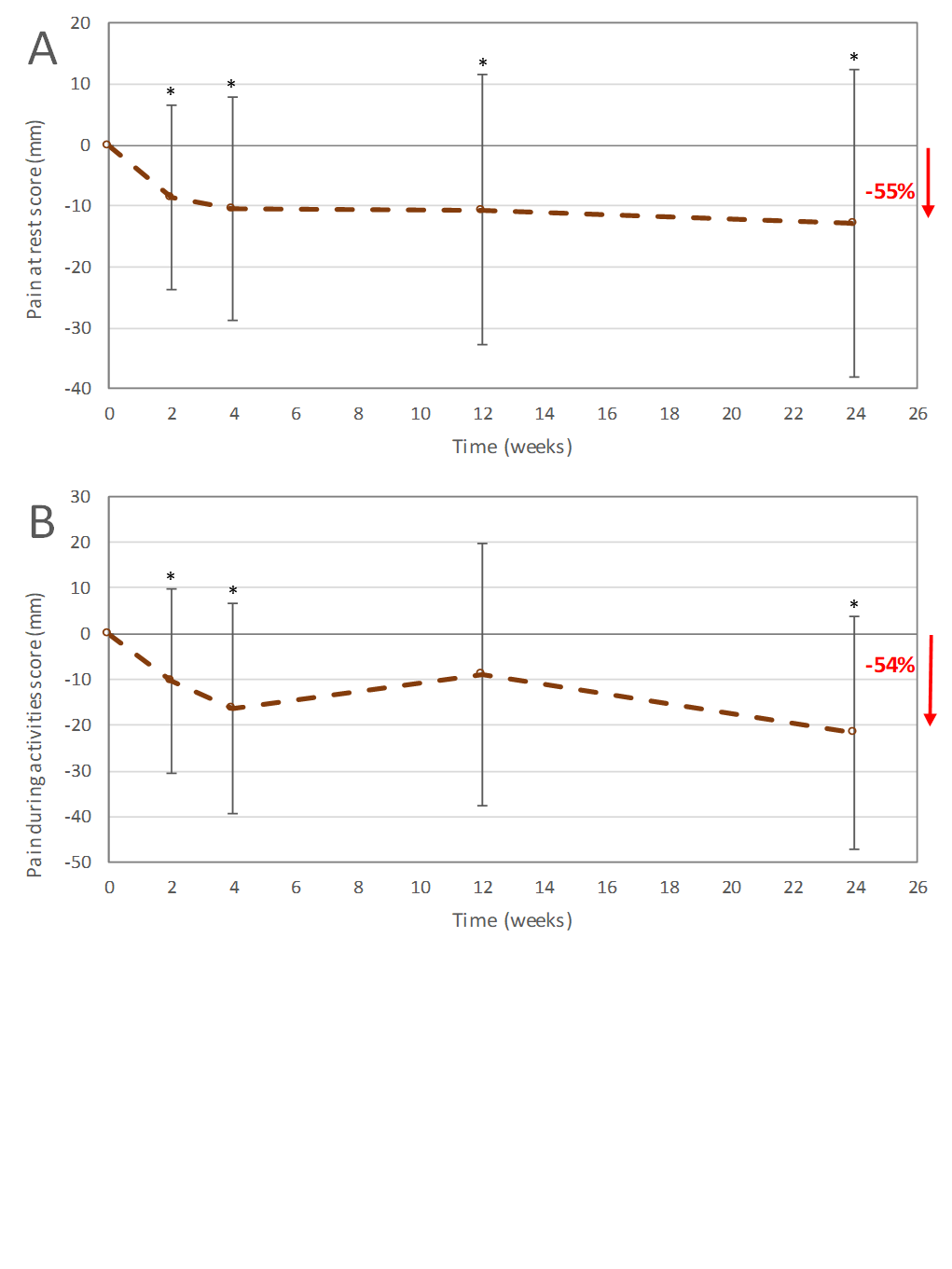

Supplement: Supplementary file 7 — Additional file 7. Change from baseline in mean (A) pain at rest score and (B) pain during activities score (per protocol efficacy population). Error bars represent the standard deviation. * significantly lower mean pain at rest/during activities score than the mean pain at rest/during activities score at baseline (least square means analysis with time and baseline as fixed effects provided p-values ≤0.05). [file 13287_2021_2432_MOESM7_ESM.png]
